# Multipath Map Method for TDOA Based Indoor Reverse Positioning System with Improved Chan-Taylor Algorithm

**DOI:** 10.3390/s20113223

**Published:** 2020-06-05

**Authors:** Cheng Hua, Kun Zhao, Danan Dong, Zhengqi Zheng, Chao Yu, Yu Zhang, Tiantian Zhao

**Affiliations:** 1 Engineering Center of SHMEC for Space Information and GNSS, East China Normal University, Shanghai 200241, China; 51181214031@stu.ecnu.edu.cn (C.H.); dndong@cs.ecnu.edu.cn (D.D.); zqzheng@ee.ecnu.edu.cn (Z.Z.); cyu@sist.ecnu.edu.cn (C.Y.); 51171214011@stu.ecnu.edu.cn (T.Z.); 2 Shanghai Key Laboratory of Multidimensional Information Processing, East China Normal University, Shanghai 200241, China; 3 Shanghai Institute of Technology, Shanghai 201418, China; yuzhang@sit.edu.cn

**Keywords:** multipath, TDOA, reverse positioning, Chan-Taylor

## Abstract

We study wireless indoor positioning systems where multiple synchronized infrastructure devices simultaneously receive signals from an object of interest whose arrival times are measured. The positioning performance is degraded by unresolvable channel multipath and non-line-of-sight (NLOS) reflctions which cause a bias in the time difference of arrival (TDOA) measurements. In order to reduce the negative effect of multi-path, a Multi-Path Map (MPM) method based on spatial domain modeling principle in the reverse positioning framework with good robustness is proposed. Meanwhile, an improved non-linear iterative algorithm with height component constrained which reduces the complexity is introduced to calculate the coordinates so that the performance of the MPM can be verified. By using the MPM measurements as pre-calibration information to compensate the TDOA observed value, the accuracy of the cooperative location based on a UWB device is 6.45 cm, which achieves 63% improvement than that of none MPM used.

## 1. Introduction

Time-of-arrival (TOA) and Time Difference of Arrival (TDOA) are two widely used measurements for outdoor precise positioning, such as the Global navigation satellite systems (GNSS). The receiver estimates the TOA of satellite signal using a sliding correlator to match the transmitted signal waveform. Then the epoch of receiver local clock corresponding to the maximum value of the correlator output is adopted as the TOA. After deducting media delays and clock errors, these TOA estimates and the knowledge of satellite locations can estimate the receiver position through a multilateration calculation. When an object has a clear line-of-sight (LOS) channel to GNSS satellites, the signal time-of-flight can be used as a proxy for the ranging distance, given the speed of light. Under the indoor environment, however, the objects are usually surrounded by clutter, the LOS channel is sometimes blocked, and the majority of the signal energy arrives through a non-line-of-sight (NLOS) reflection that bounces off surrounding obstacles multiple times. As a result, the TOA estimate can reach a bias of several hundred nanoseconds (recall that the speed of light is about 0.3 m per nasecond) compared to the LOS path. Thus, research of indoor positioning technology with multipath suppression becomes crucial. Previous positioning algorithms proposed machine learning [1,2,3,4], which used massive training data to map the relationship between received signals and positioning results. In this paper, we still inherit traditional indoor localization algorithms but consider multipath suppression from spatial domain calibration to pursue good stability and robustness for real time positioning. Research on traditional indoor positioning is mainly divided into two parts. The first part is how to obtain accurate observations through the equipment, while the second is how to calculate accurate location through a positioning algorithm [5]. Up to now, observations on indoor positioning mainly consist of Receive Signal Strength Indicator (RSSI), TOA, TDOA and angle-of-arrival (AOA) [6,7,8,9]. Among them, RSSI approach matches the energy intensity of received signal with the pre-collected fingerprint database to select the location. Thus, positioning accuracy of RSSI heavily depends on the richness of the fingerprint database and the stability of the environment. Moreover, the path loss of signal energy in the indoor environment is relatively serious, which limits the accuracy of the RSSI-method. AOA uses angle information obtained from the antenna array for positioning. As the accuracy of angle decreases rapidly (the longer the distance is, the larger the error is when at same angle), AOA is more suitable in hybrid positioning with TOA or TDOA. TOA and TDOA use time observations for positioning and the resolution of time accuracy of existing technologies can reach $10^{-18}$ s and $10^{-12}$ s for off-the-shelf commercial positioning receiver [10], so that propagation time based observation has the potential to realize precise indoor positioning. TDOA measures the TOA differences between base stations and it does not require time stamp of signal source in general positive positioning, which reduces the communication burden and improve positioning accuracy compared with TOA [11]. In this study TDOA is selected as the observations for positioning. Fang, Chan and Taylor are three popular positioning algorithms [12,13,14]. Among them, the Fang algorithm has stricter requirements on the layout of the base stations and it can only adopt three base stations, thus it lacks of redundancy of base stations for optimization in practical application scenario. Chan algorithm is a non-iterative hyperbolic algorithm through least square estimate twice, and is able to reach higher accuracy given the error obeying Gaussian distributions. However, the Chan algorithm appears vulnerable when the indoor environment contains strong multipath and NLOS scenario. Taylor algorithm is a weighted-least-squares-iteration method based on redundant observations hence it can maintain high accuracy. However, it requires precise initial value. If the offset between the initial value and the true value was too large, it may cause non-convergence. The Methods of fusing Chan and Taylor algorithms were proposed for better positioning results [15]. An improved Chan-Taylor fusion algorithm is proposed in our paper with the advantages of less computation. The remainder of this paper is organized as follows. In Section 2, TDOA error function is modeled and Multipath Map (MPM) is formed as well. Modified Chan-Taylor algorithm is introduced and combined with MPM to optimize the positioning result in Section 3. As part of experiment, robustness and availability of MPM are verified and huge amounts of experiments are proceeded with consequences in Section 4. In Section 5, we bring some instructive discussions about the advantages and the limitations of our work. In Section 6, we make a final conclusion.

## 2. System Model of Indoor Reverse Positioning and MPM Method

**Figure 1:**
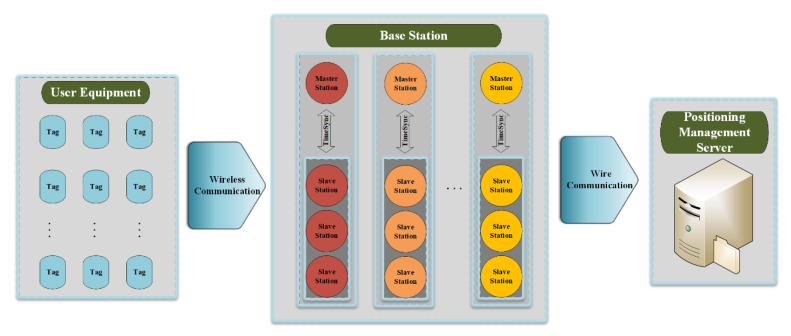
Ultra-Wideband (UWB) reverse positioning framework which contains three parts: user equipment (tags), base station (master stations and slave stations with time synchronized) and positioning management server.

We discuss two types of TDOA error modeling, that is, clock synchronization error and multipath error. Because the difference between slave base-station TOA and master base-station TOA contains error of clock bias, clock synchronization between base stations should be calibrated strictly. Solutions like synchronous package and response mode in positioning system can deal with time-sync problem effectively [16,17] suggests a pre-calibration method which can solve such task. Multipath error is caused by the superposition of direct wave and reflected waves from the wall and other indoor objects, which causes delay of received TOA. Time domain and spatial domain are two mainly research directions in terms of multipath suppression research. Research on time domain usually converts the multipath characteristics into functions independent of time. For instance, Reference [18] suggest to use Kalman Filter to deal with multipath delay; machine learning models are proposed to detect and suppress the complex multipath effect in Reference [19]; Reference [1] proposes to generate a fit distribution of multipath for optimizing TDOA measurements. In the study of space domains, the multipath characteristics of the signal transmission between transmitting end and receiving end at different spatial position are expressed and pre-calibrated in terms of time delay, and then introduced and optimized by subsequent signal processing. Our research team has proposed Multipath Hemispherical Map (MHM) as pre-calibration data in outdoor positioning [20,21], which can compensate for the error of observed quantity caused by multipath effect and achieve good results. We study space domain multipath suppression method in this paper, and verify stability and applicability of multipath error in indoor environment. We use the reverse positioning system model as shown in Figure 1 and describe the method to construct MPM. The reverse positioning system consists of fixed receiving part, mobile transmitting part and local server, which means the tags become the transmitters and base stations become the receivers during the one-way signal transmission. By using both the pre-calibration method to optimize TDOA and the modified Chan-Taylor algorithm, good experimental results are obtained with Ultra-Wideband (UWB) indoor positioning network.

The reasons for using UWB reverse positioning framework in our research are: (1) The pre-calibrated multipath error values and positioning calculation can be stored and carried out in local server much efficiently; (2) Under the reverse positioning framework, the base stations (fixed end) are considered as the receiving part and the tags (mobile end) as the transmitting part. On the premise of a relatively stable indoor environment, multipath environment of both receiving part and sending part is only related to relative space position. Because of the fixed end on the ceiling, change of the multipath only depends on the mobile end on the ground. Therefore, MPM can be drawn for each base station. Due to varying MPM in physical spaces, a virtual positioning area is assumed for building MPM.

For TOA value $\Delta t_{T}^{A_{i}}$ between base station $A_{i}$ and tag T, the observation equation is built as

(1) $$\Delta t_{T}^{A_{i}}=\frac{∥{\vec{x}}^{A_{i}}-{\vec{x}}_{T}∥}{c}+\left(\tau^{A_{i}}-\tau_{T}\right)+\tau_{m}^{A_{i}}+\varepsilon ,$$

where $\frac{∥{\vec{x}}^{A_{i}}-{\vec{x}}_{T}∥}{c}$ is the propagation time from the distance between base station $A_{i}$ and tag T, $\tau^{A_{i}}$ and $\tau_{T}$ are the clock offsets of base station $A_{i}$ and tag T respectively. $\tau_{m}^{A_{i}}$ is multipath error and ε is random error. In this paper,$A_{1}$ is assumed to be the master base station, thus TDOA observation equation between slave base station $A_{i}$ and master base station $A_{1}$ is written as

(2) $$\Delta t_{T}^{A_{i}-1}=\left(\frac{∥{\vec{x}}^{A_{i}}-{\vec{x}}_{T}∥}{c}-\frac{∥{\vec{x}}^{A_{1}}-{\vec{x}}_{T}∥}{c}\right)+\left(\tau^{A_{i}}-\tau^{A_{1}}\right)+\left(\tau_{m}^{A_{i}}-\tau_{m}^{A_{1}}\right)+\varepsilon_{i-1},$$

where $\tau_{T}$ is eliminated via TDOA, fixed ending clock errors can be compensated through synchronous package. We ignore random error sources. Thus, for TDOA observation with known locations of tag and base station, its multipath error term can be obtained by equation

(3) $$\left(\tau_{m}^{A_{i}}-\tau_{m}^{A_{1}}\right)=\Delta t_{T}^{A_{i}-1}-\left(\frac{∥{\vec{x}}^{A_{i}}-{\vec{x}}_{T}∥}{c}-\frac{∥{\vec{x}}^{A_{1}}-{\vec{x}}_{T}∥}{c}\right)-\left(\tau^{A_{i}}-\tau^{A_{1}}\right)-\varepsilon_{i-1}.$$

**Figure 2:**
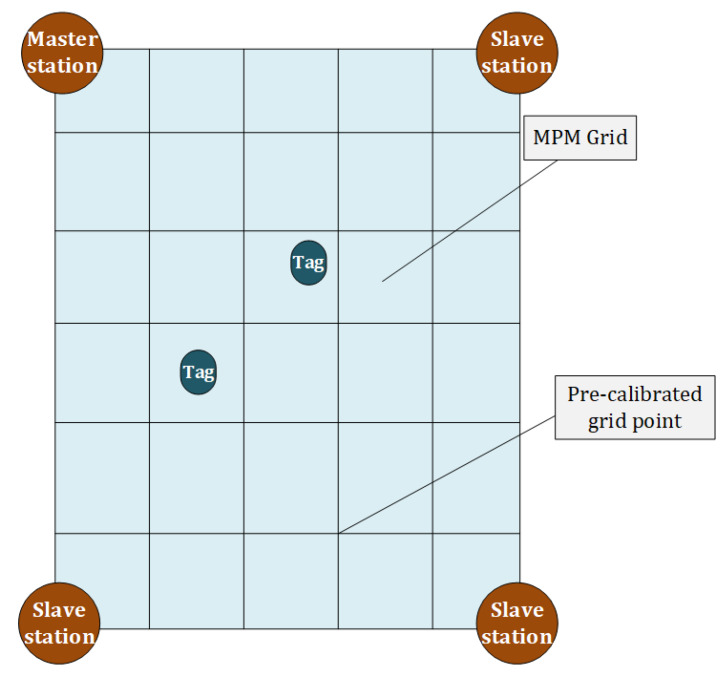
Rudiment of Multi-Path Map (MPM).

In this case, terms on the right side of the Equation (3) are all known. Multiply both sides of Equation (3) by the speed of light c, then TDOA error in form of distance is

(4) $$\left(\tau_{m}^{A_{i}}-\tau_{m}^{A_{1}}\right)\cdot c=\Delta \rho_{T}^{A_{i}-1}-d_{T}^{A_{i}-1}-\left(\tau^{A_{i}}-\tau^{A_{1}}\right)\cdot c-\varepsilon_{i-1},$$

where $\rho_{T}^{A_{i}-1}$ is the TDOA difference between base station $A_{i}$, $A_{1}$ and tag T. Multipath error is expressed as $\rho_{m}^{A_{i}-1}=\left(\tau_{m}^{A_{i}}-\tau_{m}^{A_{1}}\right)\cdot c$. When indoor environment remains unchanged, the multipath characteristics of the corresponding environment are also unchanged in spatial domain. Given that the locations of the base stations are fixed and known, and the clock offsets of base stations can be calibrated, the multipath effects can be expressed by a table of corresponding tag locations, which is an invariant map of multipath for stable environment. The nature of such a multipath map is the differential multipath effects between master base station and other base stations. We establish such multipath maps for each base station – master base station pair in form of range errors. These multipath maps are pre-calibrated and stored in server data base for TDOA observation correction. We divide the indoor area into rectangular cells, taking each grid point as the pre-calibration point. From each pre-calibration point, we collect TDOA observations with sufficient redundancy. Using Equation (4), these $\rho_{m}^{A_{i}-1}$ are calculated and their statistical average is stored in local server to build the pre-calibrated multipath values at each grid. Notice that MPM sets out from the grid positions at the receiving end, thus a TDOA MPM can be constructed from the interpolation of the pre-calibrated MPM of 4 surrounding grids as shown in Figure 2.

## 3. Improved Chan-Taylor Algorithm with MPM

This section is divided into two parts. Firstly, improved Chan-Taylor algorithm for calculating the initial coordinate will be introduced. Secondly, we expound how to utilize the MPM to calculate positioning coordinates. In real positioning scenario, the base stations are prone to be arranged in the corners of the roof, usually such an arrangement makes the heights of base stations almost the same. Based on the theory of Geometry Dilution Of Precision (GDOP), such a setting will generate almost zero resolution for height estimate. However, the accuracy of horizontal position is justified if the horizontal distribution of base stations covers surrounding directions. Thus 2-D positioning (assign a fixed height value) algorithm are commonly used in many applications. In real positioning, however, the height of tag is usually not the same as the assigned value, furthermore, the tag’s height hardly keeps the same, which brings errors to the solutions of 2-D positioning algorithm. In this paper we propose an improved Chan-Taylor algorithm with height component constrained. The TDOA observations are assumed to be time-synchronized for the clocks of base stations. Chan algorithm converts the nonlinear TDOA observation equation into form of linearized matrix, and its solution is able to provide reasonable initial value for Taylor algorithm. Therefore it prevents the solution of Taylor algorithm from non-convergence. distance between base station $A_{i}$ and tag T can be expressed as

(5) $$\rho_{T}^{A_{i}}=\sqrt{{\left(x^{A_{i}}-x_{T}\right)}^{2}+{\left(y^{A_{i}}-y_{T}\right)}^{2}+{\left(z^{A_{i}}-z_{T}\right)}^{2}}.$$

Square both sides of (5) and it becomes

(6) $${\left(\rho_{T}^{A_{i}}\right)}^{2}={\left(x^{A_{i}}\right)}^{2}+{\left(x_{T}\right)}^{2}+{\left(y^{A_{i}}\right)}^{2}+{\left(y_{T}\right)}^{2}+{\left(z^{A_{i}}\right)}^{2}+{\left(z_{T}\right)}^{2}-2x^{A_{i}}\cdot x_{T}-2y^{A_{i}}\cdot y_{T}-2z^{A_{i}}\cdot z_{T}.$$

Meanwhile, the difference between master base station $A_{i}$ and tag T can be expressed as

(7) $${\left(\rho_{T}^{A_{1}}\right)}^{2}={\left(x^{A_{1}}\right)}^{2}+{\left(x_{T}\right)}^{2}+{\left(y^{A_{1}}\right)}^{2}+{\left(y_{T}\right)}^{2}+{\left(z^{A_{1}}\right)}^{2}+{\left(z_{T}\right)}^{2}-2x^{A_{1}}\cdot x_{T}-2y^{A_{1}}\cdot y_{T}-2z^{A_{1}}\cdot z_{T}.$$

Minus (7) by (6), we obtain:

(8) $$\begin{array}{cc} \left(x^{A_{i}}-x^{A_{1}}\right) & x_{T}+\left(y^{A_{i}}-y^{A_{1}}\right)y_{T}+\left(z^{A_{i}}-z^{A_{1}}\right)z_{T}=\\ \; & \frac{1}{2}\left[\left({\left(\rho_{T}^{A_{1}}\right)}^{2}-{\left(\rho_{T}^{A_{i}}\right)}^{2}\right)+\left({\left(x^{A_{i}}\right)}^{2}-{\left(x^{A_{1}}\right)}^{2}+{\left(y^{A_{i}}\right)}^{2}-{\left(y^{A_{1}}\right)}^{2}+{\left(z^{A_{i}}\right)}^{2}-{\left(z^{A_{1}}\right)}^{2}\right)\right]. \end{array}$$

In Equation (8) when base stations are assumed having the same height, that is, $z^{A_{i}}=z^{A_{1}}$, the vertical component in $\rho_{T}^{A_{i}}$ and $\rho_{T}^{A_{1}}$ are canceled as below

(9) $$\begin{array}{cc} \left(x^{A_{i}}-x^{A_{1}}\right) & x_{T}+\left(y^{A_{i}}-y^{A_{1}}\right)y_{T}=\\ \; & \frac{1}{2}\left[\left({\left(\rho_{T}^{A_{1}}\right)}^{2}-{\left(\rho_{T}^{A_{i}}\right)}^{2}\right)+\left({\left(x^{A_{i}}\right)}^{2}-{\left(x^{A_{1}}\right)}^{2}+{\left(y^{A_{i}}\right)}^{2}-{\left(y^{A_{1}}\right)}^{2}\right)\right]. \end{array}$$

For multiple $A_{i}$ (i>3), Equation (9) leads to least square estimates [13], which are expressed in matrix form as:

(10) $$\hat{Z}={\left(G^{T}Q^{-1}G\right)}^{-1}G^{T}Q^{-1}h,$$

where $\hat{Z}={\left[{\hat{x}}_{T},{\hat{y}}_{T},\rho_{T}^{A_{1}}\right]}^{T}$ is the estimates of unknown variables; Q is the covariance matrix of observations; $G=\left[\begin{array}{ccc} x^{A_{2}}-x^{A_{1}} & y^{A_{2}}-y^{A_{1}} & \Delta \rho_{T^{\prime }}^{A_{2-1}}\\ x^{A_{3}}-x^{A_{1}} & y^{A_{3}}-y^{A_{1}} & \Delta \rho_{T}^{A_{3-1}}\\ \vdots & \vdots & \vdots \\ x^{A_{i}}-x^{A_{1}} & y^{A_{i}}-y^{A_{1}} & \Delta \rho_{T}^{A_{i-1}} \end{array}\right]$,

$$h=\left[\begin{array}{c} -{\left(\Delta \rho_{T}^{A_{2-1}}\right)}^{2}+{\left(x^{A_{2}}\right)}^{2}+{\left(y^{A_{2}}\right)}^{2}-{\left(x^{A_{1}}\right)}^{2}+{\left(y^{A_{1}}\right)}^{2}\\ -{\left(\Delta \rho_{T}^{A_{3-1}}\right)}^{2}+{\left(x^{A_{3}}\right)}^{2}+{\left(y^{A_{3}}\right)}^{2}-{\left(x^{A_{1}}\right)}^{2}+{\left(y^{A_{1}}\right)}^{2}\\ \vdots \\ -{\left(\Delta \rho_{T}^{A_{i-1}}\right)}^{2}+{\left(x^{A_{i}}\right)}^{2}+{\left(y^{A_{i}}\right)}^{2}-{\left(x^{A_{1}}\right)}^{2}+{\left(y^{A_{1}}\right)}^{2} \end{array}\right].$$

In Chan-Taylor algorithm [15], an extra weighted least squares(WLS) opeartion is added for further decreasing the perturbation errors of ${\hat{x}}_{T},{\hat{y}}_{T}$ and $\rho_{T}^{A_{1}}$ in $\hat{Z}$. In our improved Chan-Taylor algorithm, we directly use $\left({\hat{x}}_{T},{\hat{y}}_{T}\right)$ in $\hat{Z}$ as the initial value of Taylor algorithm. The reason of such change is that for Chan-Taylor algorithm, the primary purpose of Chan algorithm is to provide a better initial value for Taylor algorithm. the improved Chan algorithm also meets this requirement and the calculation amount is smaller than the previous Chan algorithm because one WLS operation is removed. In Taylor algorithm, the height of tag $z_{T}$ is constrained as known constraint whose value has been collected before and the coordinates of the base station are known, only the plane position of tag is in the iteration. The geometric equation of TDOA can be written as

(11) $$\rho_{T}^{A_{i}}-\rho_{T}^{A_{1}}=\sqrt{{\left(x^{A_{i}}-x_{T}\right)}^{2}+{\left(y^{A_{i}}-y_{T}\right)}^{2}+{\left(z^{A_{i}}-z_{T}\right)}^{2}}-\sqrt{{\left(x^{A_{1}}-x_{T}\right)}^{2}+{\left(y^{A_{1}}-y_{T}\right)}^{2}+{\left(z^{A_{1}}-z_{T}\right)}^{2}}.$$

Implement Taylor series expansion of (11) at point $\left({\hat{x}}_{T},{\hat{y}}_{T}\right)$ and solve the partial LS solution of equations of TDOA observations, through threshold iteration to optimize the calculation of positioning coordinate, then the positioning result can be obtained. Such method can both meet the requirements of actual positioning environment of LBS and avoid the error caused by vertical precision. Through improved Chan-Taylor algorithm, a calculated coordinate ${\hat{T}}_{0}\left({\hat{x}}_{0},{\hat{y}}_{0}\right)$ can be obtained. Meanwhile, we should pay attention that multipath error is still contained in the TDOA observations. Then the MPM stored in local server is introduced for ensuring the grid (namely grid G) wherein the initial location of ${\hat{T}}_{0}$ is. The multipath error of each vertex of G has been stored in the server, and for more precise multipath error, interpolation can be used to obtain the proximate multipath error of the initial location of ${\hat{T}}_{0}$. For 2-D MPM, bilinear interpolation method is more suitable. However, it should be noticed that the proximate multipath error is based on the initial location of tag, whose TDOA observations still contain the multipath error. So, one-time interpolation is not sufficient for multipath error is contained in the calculation of initial coordinates. Therefore, we propose an iterated-interpolation-TDOA-optimized algorithm in order to make the TDOA close to the truth-value, pseudocode of the algorithm is as Algorithm 1: Through such iterative algorithm, most of the multipath errors can be eliminated, and the position accuracy can be improved, experimental data will be shown in next section.
**Algorithm 1** Optimization of TDOA based on MPM.**Input:** MPM, initial coordinate, $T_{0}\left(x_{0},y_{0}\right)$, error threshold, $T_{e}$;**Output:** Final positioning coordinate, T(x,y); 1: n←0; 2: **repeat** 3:  Get multipath error ${\left(\rho_{m}^{A_{i}-1}\right)}_{n}$ by substituting $T_{n}$ into MPM; 4:  ${\left(\rho^{A_{i}-1}\right)}_{n+1}={\left(\rho^{A_{i}-1}\right)}_{n}-{\left(\rho_{m}^{A_{i}-1}\right)}_{n}$; 5:  Obtain $T_{n+1}\left(x_{n+1},y_{n+1}\right)$ by substituting ${\left(\rho^{A_{i}-1}\right)}_{n}$ into improved Chan-Taylor algorithm; 6: **until**
$|T_{n+1}-T_{n}|>T_{e}$; 7: $T\leftarrow T_{n+1}$; 8: **return**
*T*;

## 4. Experiment Results

In this section, the applicability and robustness of MPM in indoor environment are explored. Next, comparison results in terms of both accuracy and complexity between Improved Chan-Taylor algorithm and Chan-Taylor algorithm are shown. And then, the improvement of accuracy through using MPM combined with improved Chan-Taylor algorithm are discussed.

### 4.1. Introduction to Experimental Environment

**Figure 3:**
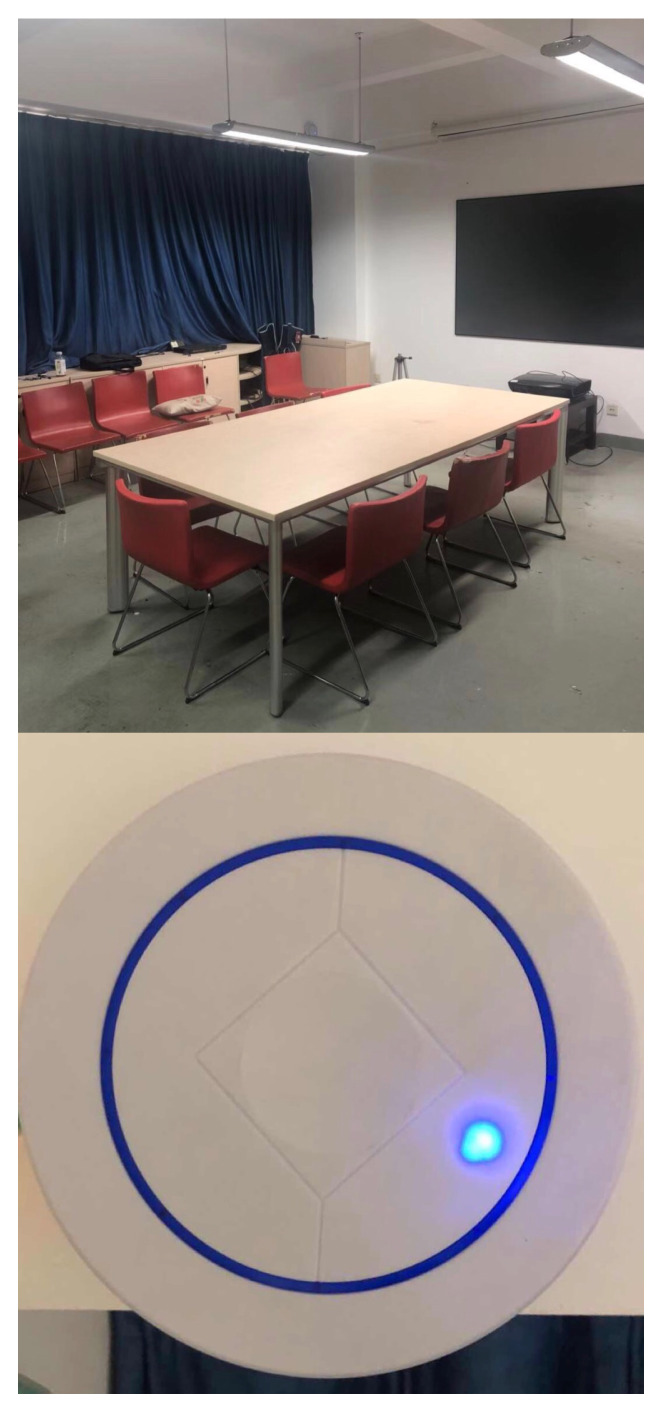
Picture of the experimental area and UWB base station.

**Figure 4:**
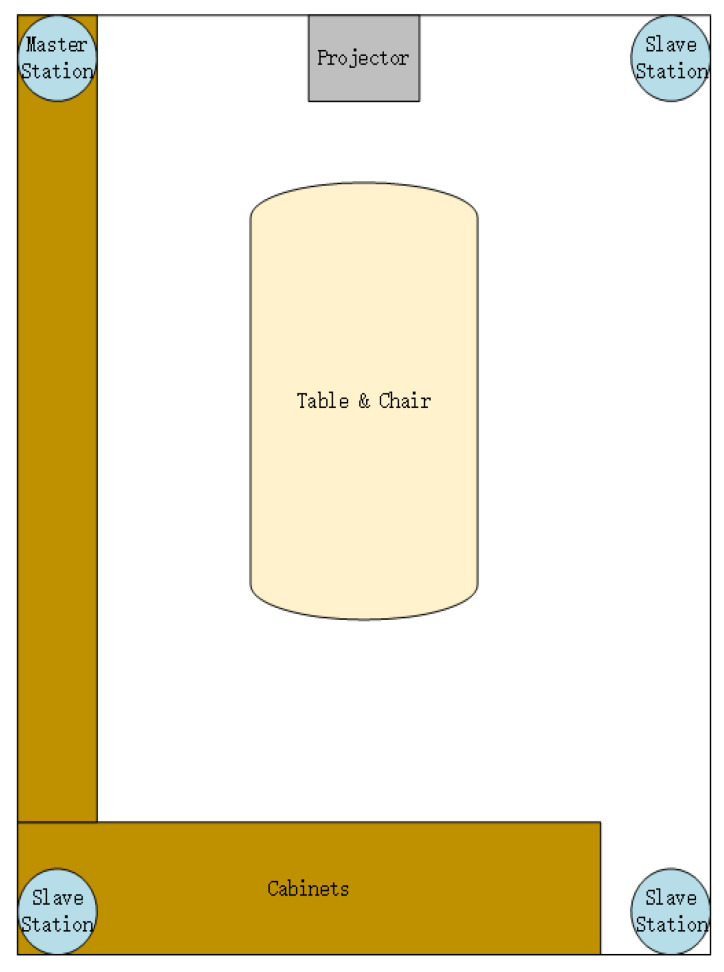
Plan of experimental area.

The experimental area is a typical meeting-room environment in the fourth floor of a building of the East China Normal University, as shown in upper half of Figure 3, with corresponding plan shown in Figure 4. The grid size is set as 0.6 × 0.8 (m${}^{2}$). The UWB system under the reverse-positioning framework used in the experiment comes from Jiangsu Tangen Technology Co., Ltd. The UWB devices consists of base stations and tags communicated wirelessly with each other based on DW1000 chips and a local service which is connected with base stations via cable, from where we get the TDOA data. An actual figure of UWB base station is shown in half bottom of Figure 3. The number of base station is four, being arranged at the same height and using structure of one master base station with three slave base stations. In this experiment, we assumed that the time-sync error has been removed through the synchronous package in the system.

### 4.2. Robustness of MPM in Non-Disturbed Environment

**Figure 5:**
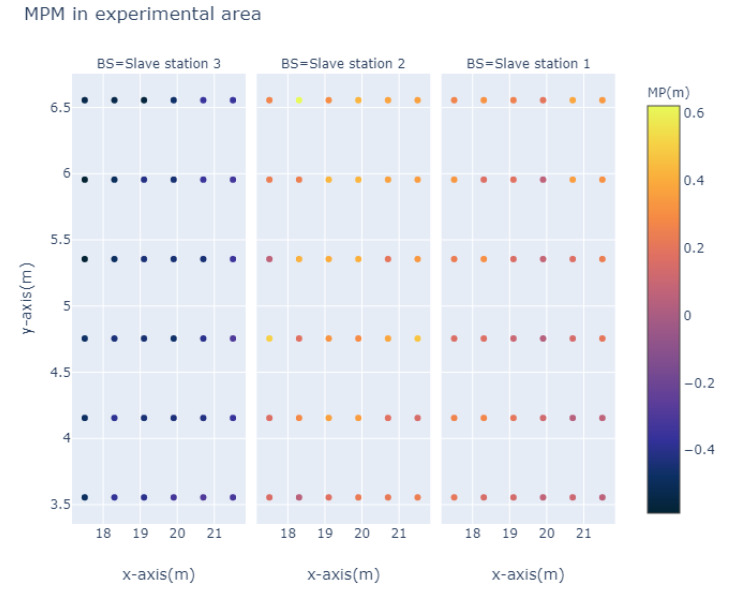
MPM Grids.

We use 3000 groups of TDOA data of each grid point to build MPM of the experimental area, and the MPM of each set of master-slave base station can be shown as Figure 5.

**Figure 6:**
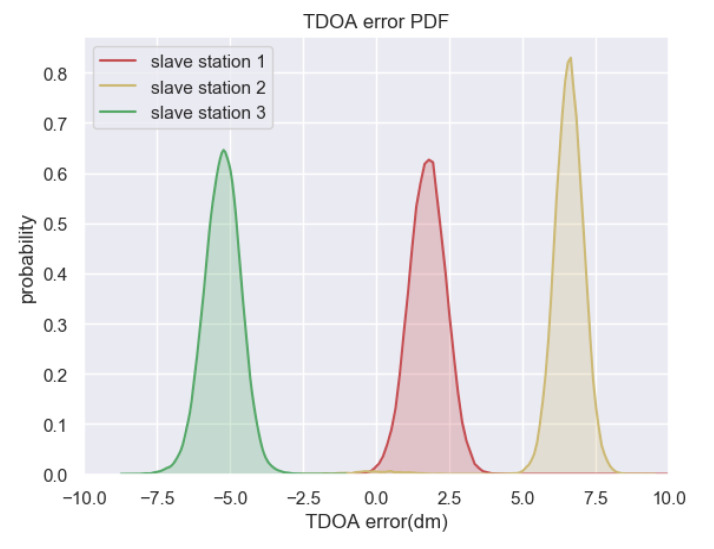
Distribution of time difference of arrival (TDOA) error in non-disturbed environment (In this figure, “dm“ means decimeter).

**Figure 7:**
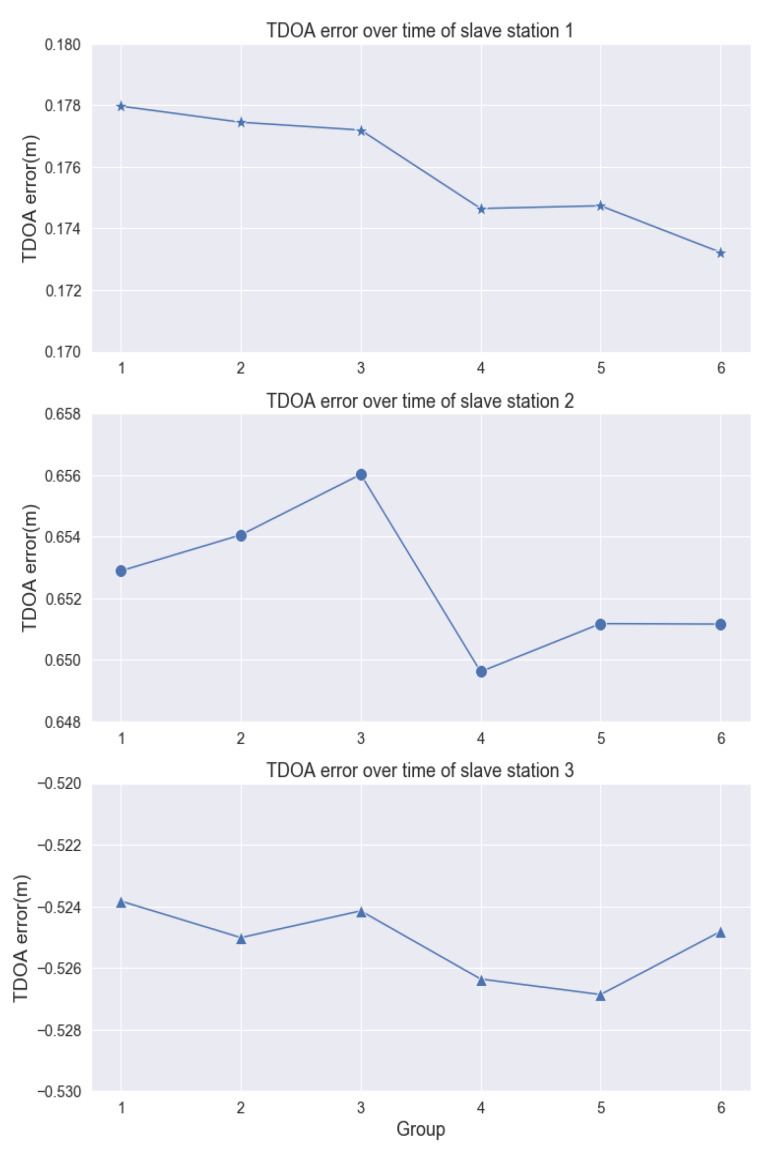
Fluctuation of the average TDOA error in non-disturbed environment.

In experiment of applicability and robustness of MPM, we select the point in experimental area with general meaning and set up the following experiments: In condition of essentially unchanged environment, 30,000 groups of TDOA observations were selected among 24-hour-continue receive and dispatch of UWB signal and calculate the multipath residuals, then we make statistical analysis figure as Figure 6 and fluctuation analysis of the average TDOA error group by time sequence as Figure 7, each group has TDOA samples in same time interval. It can be seen from the figures that the distribution curve of all the TDOA residuals of each slave base station relative to the main base station is steady, and the mean of TDOA error remain stable with only centimeter-level fluctuation. Therefore, the robustness of MPM in static environment is verified effectively.

### 4.3. Robustness of MPM in Disturbed Environment

**Figure 8:**
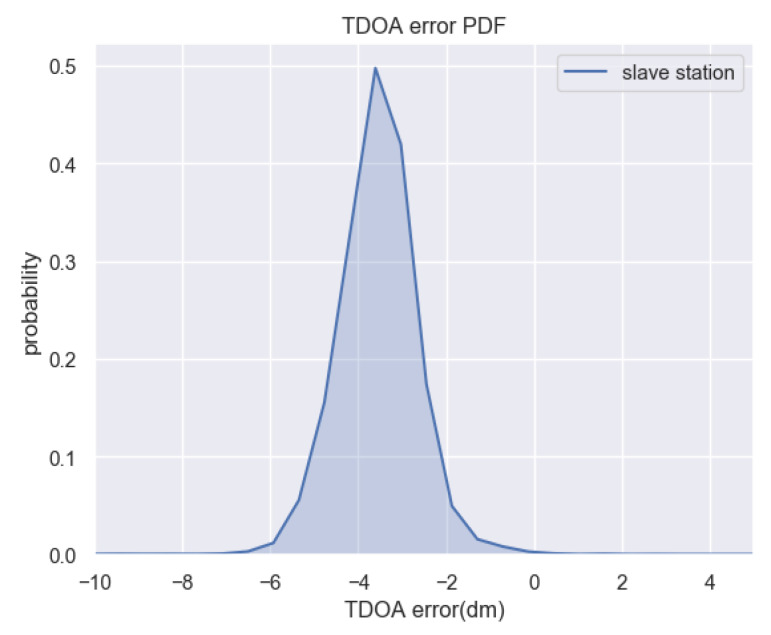
Distribution of TDOA error in disturbed environment (In this figure, “dm“ means decimeter).

**Figure 9:**
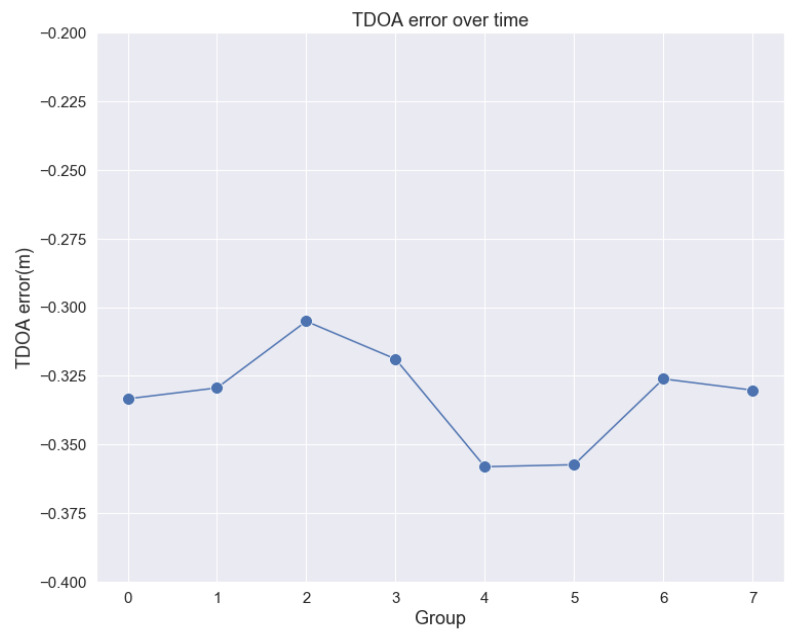
Fluctuation of the average TDOA error in disturbed environment.

In the next step, the environment of experiment area is set more complicated, we add some obstacles between the base Station and the tag, and people walking back and forth. A set of master-slave base stations is chosen and 10,000 groups of TDOA data are collected. Through analysis of the multipath error, the TDOA error PDF figure is shown as Figure 8 and fluctuation analysis of the average TDOA error group by time sequence as Figure 9. It can be seen from the figure that the distribution curve is also steady relatively, although the fluctuation of mean TDOA error becomes about 5 cm, a little larger then those in non-disturbed environment.

### 4.4. Comparison between Improved Chan-Taylor Algorithm and Chan-Taylor Algorithm

**Figure 10:**
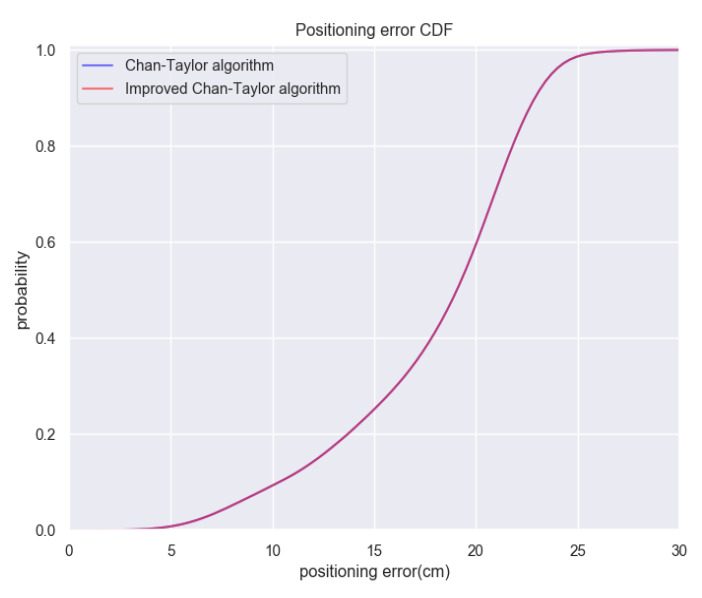
Accuracy comparison between Improved Chan-Taylor alogorithm and Chan-Taylor algorithm.

In this part, we compare Improved Chan-Taylor algorithm and Chan-Taylor algorithm in terms of both accuracy and complexity. In the accuracy part, we choose 25 observation points and implement a total of 7500 sets of TDOA observations in our experiment area. The positioning results of each sets of TDOA observations are calculated through both Improved Chan-Taylor algorithm and Chan-Taylor algorithm with vertical component constrained. Then we analyze the difference of positioning error between Improved Chan-Taylor algorithm and Chan-Taylor algorithm. The positioning error means the Euclidean distance between the real coordinate and the calculated coordinate based on corresponding set of TDOA observations. Positioning error of each group of observations is calculated and the total positioning error CDF is shown as Figure 10. In Figure 10, we can see that the accuracy of both algorithm is almost the same, it is because the Taylor algorithm is an iterative algorithm. Under the condition of convergence, the accuracy of positioning results is irrelevant to the initial value. In the complexity part, we continue using the observation data above and record the run time of calculating the position results on our computing platform based on both Improved Chan-Taylor algorithm and Chan-Taylor algorithm. The processor configuration of our computing platform is Intel Core i7-9750H @ 2.60 GHz provided by Lenovo Co., Ltd. (Beijing, China), and we use Python (Version 3.7.3) as the programming language for writing and running the code of both algorithm. We give the average running time of totally 7500 groups of calculation for both Improved Chan-Taylor algorithm and Chan-Taylor algorithm in Table 1: From Table 1, it is clearly shown that the execution speed of Improved Chan-Taylor alogorithm is faster than Chan-Taylor algorithm. To be specific, the calculate time for each run is decreased by 34 %. From the experiments above, the conclusion that Improved Chan-Taylor alogorithm has the same accuracy but less complexity than Chan-Taylor algorithm can be proved.

### 4.5. Application of MPM in Actual Positioning Scene

**Figure 11:**
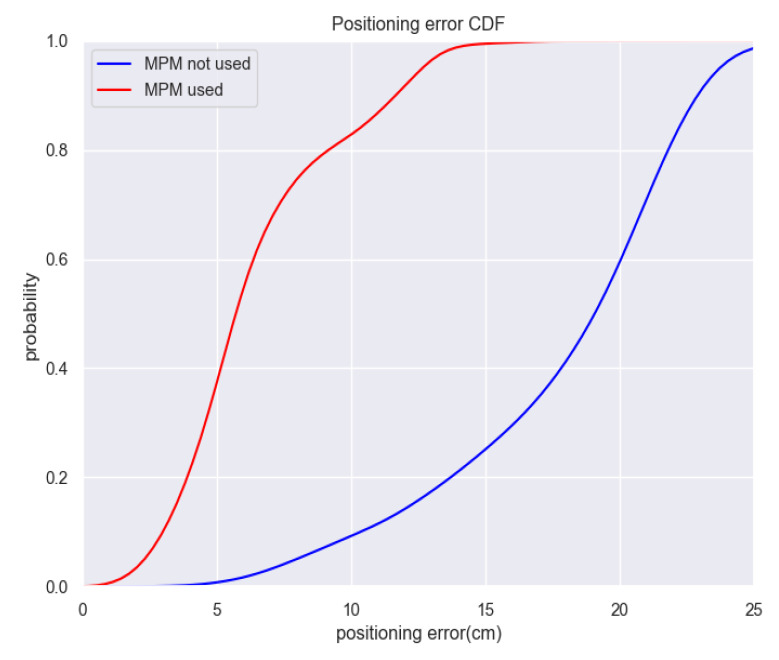
Positioning error CDF in experiment area.

In the experiments discussed the improvement of accuracy through using MPM, we choose 25 observation points and implement a total of 7500 sets of TDOA observations, which is the same as the data in Section 4.4. Then we analyze the difference of positioning error between MPM used and MPM not used based on improved Chan-Taylor algorithm. The positioning error means the Euclidean distance between the real coordinate and the calculated coordinate based on corresponding set of TDOA observations. Absolute positioning error of each group of observations is calculated and the positioning error CDF is shown as Figure 11.

As can be seen from the Figure 11, absolute positioning error used MPM is mainly under 14 cm while in the case of MPM not used, the error mainly locates in the range of 15–23 cm. By comparing root-mean-square error (R.M.S.E.), mean absolute error (M.A.E) and standard deviation (S.D.), detailed error information is contained in Table 2. From Table 2, we can get the information that utilizing MPM can improve the positioning accuracy by about 63%, it can be seen from the S.D. that the stability is also improved a little. It can be seen that MPM can effectively improve the positioning accuracy and thus provide better LBS in actual location scenarios.

## 5. Discussion

In this paper, we proposed a TDOA pre-calibration method named MPM based on the traditional algorithm. In the experiment part, we discussed the stability of MPM in the case of disturbed and non-disturbed environment respectively, the results showed that the multipath error of TDOA in each point is relatively stable, thus the idea of MPM is valid. Then we applied the MPM in actual measurement. Judging from the experimental results, the use of MPM significantly improved the accuracy of indoor positioning. Then we also proposed the improved Chan-Taylor algorithm based on the traditional Chan-Taylor algorithm with same accuracy but less computation which was proved in experiment section. The proposed MPM method enables us to implement indoor positioning with higher accuracy through certain preparations before actual positioning projects. Therefore, the MPM method is very suitable for positioning scenarios with high accuracy requirements, such as Wise Information Technology of 120, tunnel staff positioning system and so on. Meanwhile in the scenario of positioning projects with general accuracy requirement, the Improved Chan-Taylor alone can also provide higher execution efficiency of positioning calculation, which is important for real-time positioning scenarios.

Although the MPM method has enhanced accuracy in our experiments, some issues worth discussing should be mentioned. The first problem is the convergence of MPM. We need to find the compensation values of TDOA observations repeatedly through Improved Chan-Taylor algorithm thus iteration method is used in our method. However, the convergence of such iteration is hard to be proved directly. The improved Chan-Taylor algorithm can be considered as the approximation of the true localization based on the current TDOA observations, then a more precise TDOA corrections can be calculated through interpolation of the corresponding MPM grids. Through those two optimization methods, the search area is narrowed and the convergent point can be reached effectively. In the initial phase, we used a few experiments to verify the idea and found that such method can provide accelerated convergence results. Therefore, we proceed with a huge amount of observations and all the results were converged. Thus the convergence problem needs more effective explanation in the future work. The second problem is how to choose the size of MPM grid, which may cause impact on positioning accuracy. Actuary, we had made a tentative attempt of data collecting for smaller grids before. However, it seemed that in our experiment environment, the changes of multipath error among smaller grids were not obvious. Based on the experiment, we set the size of grid as 0.6 × 0.8 (m${}^{2}$), which matches our experimental environment. To maintain the accuracy of the correction model the grid size should be environment-dependent. The complex the environment is, the small size grid should be chosen.

The theoretical consideration of MPM is that under the reverse positioning system the base stations are usually mounted on stable environment, such as ceilings, where the near-field multipath effects remain unchanged, thus the main portion of multipath errors (usually near-field multipath) of each grid keeps approximately fixed value. However, when the indoor environment changes greatly, the state of multipath effect may also change. At this time, the MPM should be calibrated again to maintain the same accuracy as before.

## 6. Conclusions

The multipath effect is the bottleneck in existing high-accuracy indoor positioning. For this situation, MPM method for decreasing multipath error is proposed in our paper. As a kind of TDOA optimization method based on pre-calibration, MPM does not require complex software or hardware. For different indoor environment and layout of base stations, MPM, which explores the multipath effect in space domain, can bring more effective and reasonable process on multipath suppression. And considering the execution efficiency, Improved Chan-Taylor algorithm is proposed in our work which decreases the calculate time by 34% with almost the same accuracy as the Chan-Taylor algorithm. Then based on improved Chan-Taylor algorithm, the MPM method is used to optimize the TDOA observations and the positioning accuracy is improved by 63%. In the future work, we prepare to make further analysis on the resolution of the MPM grid, searching the balance between the positioning error and the algorithmic complexity of operation to improve the method in this paper. Interest is also on the theory proof of the convergence of MPM algorithm and the recalibration of MPM caused by huge changes of the indoor environment, hoping to give an applicable optimization scheme.

## Figures and Tables

**Table 1:** Running time of algorithm.

	Improved Chan-Taylor (ms)	Chan-Taylor (ms)
Average time	0.220	0.331

**Table 2:** Positioning error information.

	MPM Not Used (cm)	MPM Used (cm)
R.M.S.E.	18.42	7.16
M.A.E.	17.81	6.45
S.D.	4.72	3.12
